# Anaphylaxis to Polysporin® ointment in a pediatric patient

**DOI:** 10.1186/1710-1492-8-S1-A13

**Published:** 2012-11-02

**Authors:** Anna-Claire Coleman, Sandeep Kapur, Wade Watson, Lori Connors

**Affiliations:** 1 Department of Pediatrics, Dalhousie University, Halifax NS, Canada; 2 Division of Allergy, IWK Health Centre, Halifax NS, Canada; 3 Halifax Allergy and Asthma Associates, Halifax NS, Canada; 4 Department of Internal Medicine, Dalhousie University, Halifax NS, Canada

## Background

Topical antibiotics are widely used over the counter preparations to treat and prevent common skin infections. Severe allergic reactions to these preparations are rare. We report a case of anaphylaxis after topical application of Polysporin® ointment.

## Case report

A 13 year old male rubbed a white eraser against the dorsum of his hand at school and the skin became abraded with some open areas of broken skin. He applied Polysporin® ointment (polymyxin B sulfate and bacitracin) to the area after he arrived home. Within 15 minutes he developed urticaria on his back, legs and arms. His eyes became very itchy and he developed marked angioedema of his eyelids with urticaria around his eyes. He developed difficulty breathing and described a tight feeling in his chest. He took diphenhydramine and salbutamol via MDI. He did not go to the Emergency Department. His symptoms gradually resolved after approximately two hours. Previous use of Polysporin® in the past did not cause any difficulties. Epicutaneous testing was positive to a 1 in 10 dilution of Polysporin® ointment. Testing was negative to latex. Further testing to the specific components, polymyxin B sulfate and bacitracin is pending.

## Conclusion

This patient’s history and skin test results are in keeping with an IgE-mediated reaction to this Polysporin® preparation. Although rare, severe reactions can occur. Health care professionals should be reminded to inquire about previous reactions to topical antibiotics before suggesting their use.

